# Does Cattle Milieu Provide a Potential Point to Target Wild Exophilic *Anopheles arabiensis* (Diptera: Culicidae) with Entomopathogenic Fungus? A Bioinsecticide Zooprophylaxis Strategy for Vector Control

**DOI:** 10.1155/2012/280583

**Published:** 2012-08-15

**Authors:** Issa N. Lyimo, Kija R. Ng'habi, Monica W. Mpingwa, Ally A. Daraja, Dickson D. Mwasheshe, Nuru S. Nchimbi, Dickson W. Lwetoijera, Ladslaus L. Mnyone

**Affiliations:** ^1^Biomedical and Environmental Thematic Group, Ifakara Health Institute, P.O. Box 53, Off Mlabani, Ifakara, Morogoro, Tanzania; ^2^Vector Group, Liverpool School of Tropical Medicine, Liverpool L3 5QA, UK

## Abstract

*Background*. * Anopheles arabiensis * is increasingly dominating malaria transmission in Africa. The exophagy in mosquitoes threatens the effectiveness of indoor vector control strategies. This study aimed to evaluate the effectiveness of fungus against * An. arabiensis* when applied on cattle and their environments. *Methods*. Experiments were conducted under semi-field and small-scale field conditions within Kilombero valley. The semi-field reared females of 5–7 days old *An. arabiensis* were exposed to fungus-treated and untreated calf. Further, wild *An. arabiensis* were exposed to fungus-treated calves, mud-huts, and their controls. Mosquitoes were recaptured the next morning and proportion fed, infected, and survived were evaluated. Experiments were replicated three times using different individuals of calves. *Results*. A high proportion of *An. arabiensis* was fed on calves (>0.90) and become infected (0.94) while resting on fungus-treated mud walls than on other surfaces. However, fungus treatments reduced fecundity and survival of mosquitoes. *Conclusion*. This study demonstrates for the first time the potential of cattle and their milieu for controlling *An. arabiensis*. Most of *An. arabiensis* were fed and infected while resting on fungus-treated mud walls than on other surfaces. Fungus treatments reduced fecundity and survival of mosquitoes. These results suggest deployment of bioinsecticide zooprophylaxis against exophilic *An. arabiensis*.

## 1. Background

The feeding and resting behaviours of African malaria vectors are the key determinants of a high malaria transmission intensity [1]. Among African malaria vectors, *Anopheles gambiae* s.s and *An. funestus* are well adapted over many generations feeding and resting inside human houses (i.e. endophagy and endophily, resp. [1, 2]). In contrast, *Anopheles arabiensis* is opportunistically feeding on humans [3, 4] or cattle [1, 2, 5–7] and resting outside (exophily) [8] or inside (endophily) houses [3, 4], based on availability of their preferred host species. The endophilic vector species have been well controlled by insecticide-treated nets (ITNs) [9, 10], long-lasting insecticide-treated nets (LLINs) [11], and indoor residual spraying (IRS) [12, 13] than exophilic population of *An. arabiensis* in most parts of Africa. These suggest that behaviours of malaria vectors are crucially important on designing effective control strategies.

Outdoor feeding behaviour of exophilic *An. arabiensis* minimizes the risk of being killed by ITN/LLIN and/or IRS since these measures are exclusively applied indoors. A recent study predicted that these interventions increase extrinsic mortality of endophilic, anthrophilic *An. gambiae s.s,* and *An. funestus* and consequently generate selection pressures for insecticide resistance [14]. These explain the phenomena of declining endophilic vectors, increase of exophilic *An. arabiensis* in most parts of Africa [10, 11], and shift of *An. gambiae s.s* from endophagy to exophagy in some locations [15]. The widespread insecticide resistance in population of African malaria vectors [16, 17] suggests an urgent need for alternative strategies to complement the universal coverage of LLIN.

Entomopathogenic fungi of the group hyphomycetes, notably *Metarhizium anisopliae* and *Beauveria bassiana,* hold a great promise as complementary mosquito control bio-insecticides [18–23]. These are slow acting [19, 24] and nonrepellent bio-insecticides to *Anopheles* and *Culex* mosquitoes [25]. These bio-insecticides kill mosquitoes between 4–10 d after exposure [18, 26–29], before malaria parasites become transmissible as such parasites require ≥12 d to develop within a mosquito [30]. Also fungus infection reduces blood feeding propensity, life-time fecundity, flight propensity, and flight stamina [19, 27, 31, 32]. Also fungus infection inhibits development of *Plasmodium* parasites within mosquitoes [20, 32] and kills both insecticide susceptible and resistant mosquitoes [22, 33, 34]. Based on these merits, fungi provide a potential candidate for bio-insecticide zooprophylaxis against exophilic *An. arabiensis*.

However, optimal methods for delivering fungi against outdoor feeding and resting malaria vectors are yet to be developed. An effective and practical fungus delivery method requires spores to be applied on sites whilst maximizing exposure, maintaining spore viability, and minimizing the required dose of conidia. Few point source delivery methods have been tested: (a) eave curtains, baffles to target host seeking mosquitoes [35], (b) odour-baited stations, clay pots, and cotton cloth attached on the ceiling roof to target resting mosquitoes [29, 36, 37]. Only two of these methods, however, achieved high infection rates (>75% ) in a population of wild *An. arabiensis* through use of human sleepers [35], and synthetic human odours [29] to attract these exophilic, zoophilic mosquitoes. However, in rural settings, cattle, the naturally preferred hosts for exophilic *An. arabiensis,* are kept close to human houses but inside mud houses or wood posts shelters with thatched roof or palm fronds. Such environment (milieu) may favour spore viability and maintain their infectiousness against mosquitoes thereof. Equally important, this milieu may passively attract exophilic *An. arabiensis* to blood feed on their preferred cattle hosts and subsequently rest on mud walls and/or thatched roof. However, this cattle milieu has never been exploited as an option to apply fungi against exophilic *An. arabiensis*.

The purpose of the current study was to evaluate the efficacy of entomopathogenic fungus against the local population of exophilic *An. arabiensis* when applied on various delivery surfaces either individually or in combination: calf, mud walls, and cotton-cloth roofs.

## 2. Materials and Methods

### 2.1. Study Site

The study was conducted at the Ifakara Health Institute (IHI) in the Kilombero valley, south eastern Tanzania. The predominant malaria vectors in this valley include *An. gambiae s.s*, *An. funestus,* and *An. arabiensis* [38–41]. Recent field studies have indicated the decline of *An. gambiae s.s* population within Kilombero valley [11]. However, a population of exophilic *An. arabiensis* is increasing within this valley and other parts of Africa [10, 11]. The preferred hosts for this vector, cattle, are commonly kept in or near human houses within Kilombero valley [42].

### 2.2. Mosquitoes

#### 2.2.1. Semifield Reared *Anopheles arabiensis*

Semifield experiments were conducted using female *An. arabiensis* reared under semifield conditions at the IHI. The colony of *An. arabiensis* was established with individuals from the village of Sagamaganga in 2007 and 2008 and is maintained at an ambient temperature varying from 25 to 32°C and a relative humidity of 51 to 90% within the semifield system [43, 44].

#### 2.2.2. Wild Population of *Anopheles arabiensis*

Small-scale field trials were conducted against freely flying wild population of *An. gambiae s.l* at Lupiro village in Ulanga district, Kilombero valley (8.385°S and 36.670°E). The recent species identification using molecular biology techniques of polymerase chain reaction (PCR) demonstrated that 98% of *An. gambiae s.l* wild population in this village is composed of *An. arabiensis* [45]. This confirms that *An. arabiensis* is the most predominant malaria vector in this village. Generally, these mosquitoes are known to feed on cattle and rest outside human houses in most of villages within Kilombero valley [42]. 

### 2.3. Fungal Isolates, Formulation and Application

Two species of entomopathogenic fungi of the group hyphomycetes were used: *Beauveria bassiana* isolate I93-825 and *Metarhizium anisopliae* isolate IP 46. The former and the latter were used in semifield and small-scale field experiments, respectively. These experiments were intended to demonstrate delivery methods of fungus and not a comparison between species. Therefore, before each experiment, conidia viability (>85% germination on Sabouraud dextrose agar) was confirmed.

Fungal conidia were suspended in a 1 : 1 mixture of highly refined Enerpar oil (Enerpar M002, BP Southern Africa Ltd) and Shellsol oil (Shellsol T, South Africa Chemicals). The test suspensions of conidia were prepared and applied to delivery surfaces based on procedures described by Mnyone et al. [18]. For semifield experiments, a calf was treated with 23 mL suspension of 2.3 × 10^10^ conidia (1 × 10^9^ conidia mL^−1^). The calf was sprayed with the suspension of conidia using a handheld pressure sprayer (Minijet SATA, Germany) at a constant pressure of 2 bars over its whole body including tail and legs. For small-scale field trials, hut walls and cotton-cloth roof were also sprayed with 23 mL conidia suspensions (5 × 10^10^ conidia m^−2^). As in the semifield conditions, 23 mL suspension of 5 × 10^10^ conidia (2.2 × 10^9^ mL^−1^) was sprayed per calf in the small-scale field trials. The control hut and calves were treated with equal volumes of oil mixture alone. Treatments were done at the study site under tree shade to avoid the effect of intense sunlight on conidia. All surfaces, except cottoncloth, were treated 5 h prior to the experiments to allow proper drying. Cottoncloth was treated 24 h a prior. Calves were restrained and left to dry under tree shade.

### 2.4. Experimental Setup and Design

#### 2.4.1. Net Huts

**Figure 1:**
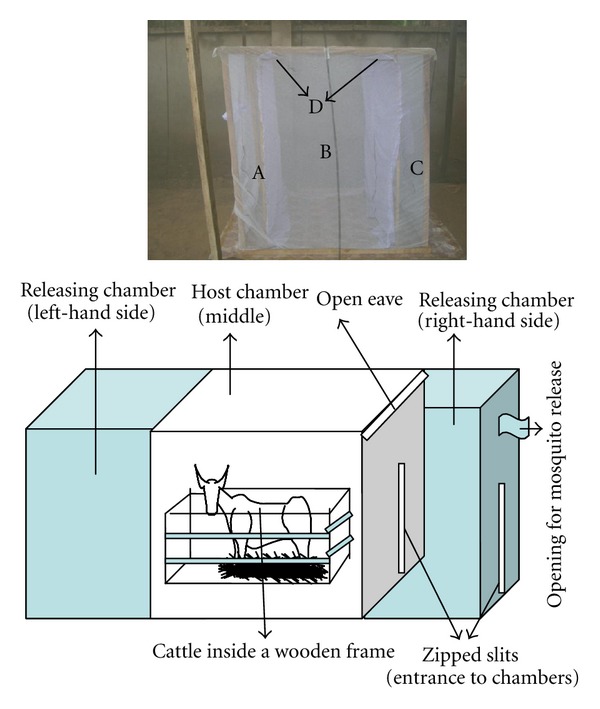
Picture of a rectangular net hut and its schematic representation. The sections of net hut are (A) left-hand side releasing chamber, (B) host or middle chamber, (C) right-hand side releasing chamber, and (D) open eaves with baffles to allow host-seeking mosquitoes to enter host chamber.

The rectangular net hut (1.5 × 1.8 × 2.1 m, Figure 1) was constructed from a regular bed net (Safi net) and placed within a netting enclosed tunnel (100 × 3.5 × 2.70 m) of the IHI semifield system. The rectangular shape was maintained by fixing wooden rectangular frame from inside the bed net. The rectangular net hut was partitioned into three chambers using pieces of white clothes: chamber 1 on the left-hand side, chamber 2 on the right-hand side (2 releasing chambers), and a middle chamber (a host chamber). Releasing chambers had a round opening with a sleeve through which mosquitoes were introduced. The top of the vertical white clothes partitioning releasing chambers from host chamber were slanted towards the middle chamber to form baffles with open eaves of 1.5 cm. These openings between top side of a net and the vertical white cloth mimic open eaves that allowed mosquitoes from the releasing chamber to enter the host chamber. Releasing chambers had two zipped slits. The first slit formed entrance into the releasing chamber from outside and the second slit allowed entering into the host chamber from releasing chamber. These slits also allowed introducing a calf into the host chamber. In this chamber, there was a small wooden cage (1.10 × 0.59 × 1 m) for restraining a calf not to damage the net hut. The floor of the net hut was made of nylon carpet for easy cleaning of calf urine and faeces and observing for dead mosquitoes. A strip of grease was kept on the nylon carpet surrounding the net hut from outside to ensure no ants enter the hut and eat (scavenging on) dead mosquitoes.

#### 2.4.2. Mud Huts

**Figure 2:**
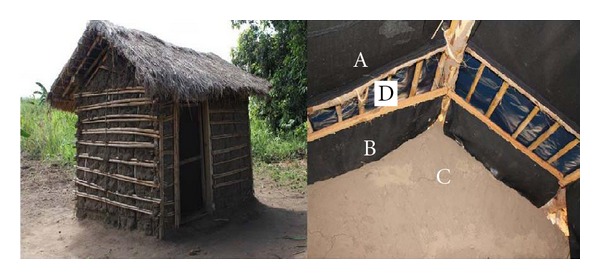
Mud hut at Lupiro village with fixed (A) cotton-cloth roof treated with fungal conidia, (B) baffles to reduce exit of mosquitoes, (C) mud walls either treated with fungal conidia or untreated for resting mosquitoes, and (D) open eave to allow host seeking mosquitoes to enter inside the hut.

Mud huts (2.2 × 1.6 × 1.77 m, Figure 2) were constructed in the same way people are building their local houses at Lupiro village. Hut walls were made from bamboo sticks and soil from the same village. The walls were plastered by mud. The roofs were made from thatches. The space between the roof and wall was 14 cm from outside. The baffles towards inside the hut were constructed to progressively reduce an eave of 14 cm to 3 cm. This tapered eave space allowed host-seeking mosquitoes to enter the hut but preventing them from exiting through hut. Therefore, wild population of *An. arabiensis* could be attracted to feed on a calf inside the hut and rest on mud plastered walls and/or cotton-cloth attached on thatched roofs.

#### 2.4.3. Experimental Procedures

The semifield experiments were conducted following 2 × 2 Latin square design (LSD) in two rectangular net huts constructed within a unique Ifakara tunnel system. Two calves were randomly selected from Ifakara communities. One calf was treated with fungal conidia and the second calf remained untreated (control). One net hut contained fungal-treated calf (treatment) and the other hut contained untreated calf. These calves were introduced inside the host chamber in the evening (6:30 pm). Then 150–200 female *An. arabiensis* were introduced into each releasing chamber and left to forage overnight on the calf by entering through open eaves as they do in the natural environment. The next morning all mosquitoes were recaptured from releasing and host chambers. The recaptured mosquitoes were identified whether fed or unfed and then held in the semifield insectary to monitor for their subsequent fitness traits including longer-term survival and fecundity. The number of eggs and days survived were counted and recorded. Dead mosquitoes were put onto moist filter paper in petri dishes, sealed with parafilm, and incubated inside a humid chamber for 3–5 d, after which they were examined for fungal sporulation. The efficacy of fungus over time (persistence) was also preliminarily assessed by exposing mosquitoes to fungal-treated calves at 3 d after treatment. These experiments were replicated four times using four different individual calves.

The small-scale experiments were conducted following 3 × 3 LSD in three mud huts constructed at the periphery of the Lupiro village. Two mud huts were treated with fungal conidia (one was treated on the walls and the other on the roof) and one hut was left untreated. Three calves were randomly selected from the local communities. Only one of these calves was treated with fungal conidia and introduced inside untreated hut, while the other two untreated calves were placed inside wall- and roof-treated huts, respectively. These calves were introduced into the mud huts at evening time (6:00 pm) and left there overnight. The next morning, all mosquitoes were recaptured from inside each mud hut. The mosquitoes were identified whether fed or unfed. All these mosquitoes were individually transferred into a paper cup and held in the field insectary to monitor their survival. Also all blood fed mosquitoes were provided with wet filter paper in the paper cup for them to lay eggs at 4 d after blood feeding on calf (i.e. *Anopheles* mosquitoes develop eggs within 3-4 days after blood feeding [46]). The number of eggs and days survived was recorded. Also mosquitoes were processed for sporulation as in the semifield system. The calves were rotated between huts to control for the variation between huts. Therefore, the calves spent three days in the same hut before shifting into another hut. These experiments were replicated three times using three different individual calves to control the variation between their in attractiveness.

### 2.5. Statistical Analyses

Statistical analysis was conducted to evaluate effects of three fungus delivery methods (cattle, mud walls, and cotton-cloth roofs) against a population of exophilic *An. arabiensis*. Three key parameters were analysed: proportion of fed mosquitoes (feeding success) and showing fungal outgrowth (sporulation), number of eggs (fecundity), and postexposure survival of mosquitoes (number of days survived). The first two parameters are binomial response variable, whereas the last two are continuous response variables.

The binomial and continuous response variables were analysed using generalized linear mixed effect models using an appropriate link function in the *R* statistical package [47], with “treatments” as fixed effects and “replicate” as a random effect. A base model including only the random effect of “replicate” was constructed, to which the main effect of “treatment” was added to form a full model. The significance of additional fixed effects of treatments was tested by sequentially adding this term to a base model and applying likelihood-ratio test (LRTs) to test if they led to a significant improvement (*P* < 0.05). For semifield experiments where fungus-treated was compared with untreated calf, the chi-square (*χ* ^2^) generated from model comparisons was used to test for significant differences between control and treatment. Whereas for small-scale experiments, more than two treatments were compared and when treatment was identified as being statistically significant, then Tukey's post hoc test (adjusting for multiple comparisons) was used to identify significant two-way differences between control and treatments and within treatments using *z*-values. All *z*-values reported small-scale experiments are those generated from multiple comparisons in *R* statistical software [47].

The continuous response variable of survival data (only infected mosquitoes noninfected were excluded from analysis) are rarely normally distributed and thus were appropriately analysed using Cox proportional hazards model (coxph) [47, 48]. This model tested whether survival of the mosquitoes varies between delivery methods (calf, house wall, and roof) and days after treatment. A frailty function was used to incorporate the random effect of “replicate”, and “treatments” were fit as main effect in *R* statistical software [21]. The coxph model compared the survival curves of different treatments and gave statistical significant differences in overall mortality rates in hazard ratio (HR) values, which indicate the daily risk of dying [48]. These hazard ratios have now replaced direct comparison of mortality rates after a specific point in time using *t*-tests [49–51]. An HR value of 1 indicates equal mortality rates between treatments and control, an HR value > 1 indicates significantly greater mortality rates in treatment than control, and HR < 1 indicates significantly lower overall mortality rates in treatment than control.

## 3. Results

A total of 1,690 and 547 female *An. arabiensis* were attracted to calf and being recaptured from, respectively inside net huts in the semifield and mud huts in the field experiments. Almost all recaptured mosquitoes were blood fed (a proportion of 0.90 to 0.99) under both semifield and field conditions. This study evaluated the effects of fungus delivery options (calf, mud wall, and cotton-cloth roofs) on infection rates (sporulation), fecundity, and survival of blood fed, exophilic *An. arabiensis*. 

### 3.1. Semifield Experiments

**(i) Proportion of Fed Mosquitoes**

**Figure 3:**
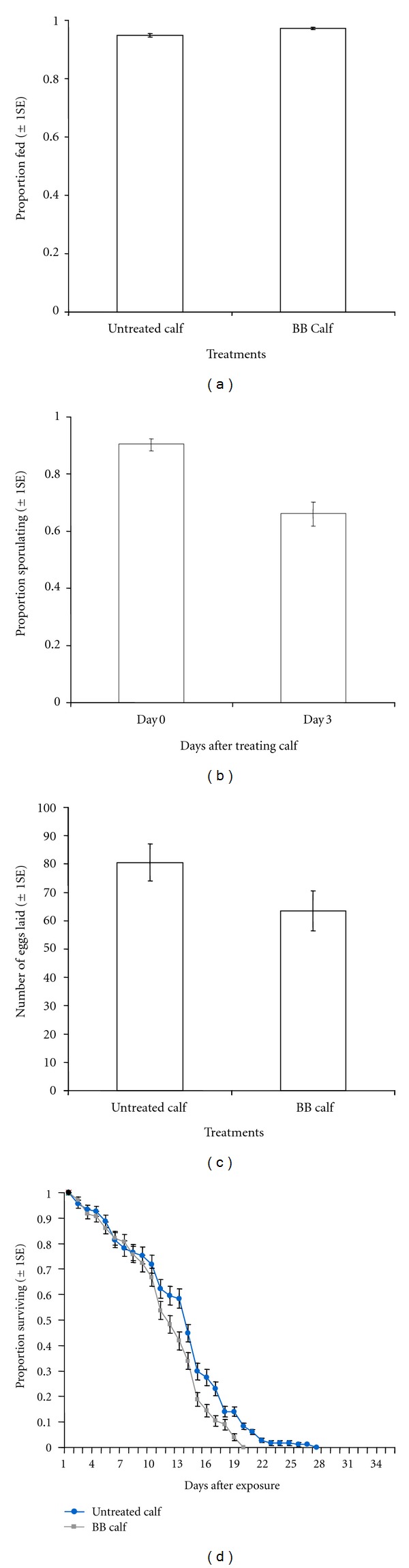
Effects of *B. bassiana* against semifield reared exophilic *An. arabiensis* populations: (a) Estimated proportion (±  1s.e) of fed after exposure to fungus-treated and untreated calf, BB calf indicates a calf sprayed with conidia suspension of *Beauvaria bassiana.* (b) Estimated proportion (±  1s.e) of infected mosquitoes after exposure to fungus treated calf on 0 d and 3 d. (c) Estimates (±  1s.e) of the mean number of eggs laid by mosquitoes after exposure to fungus-treated and untreated calf. (d) Survival of mosquitoes after exposure to fungus-treated and untreated calf, the lines represent the survival function as estimated from fitting Cox proportional hazard model (controlling for random variation between individual calves).

The proportion of *An. arabiensis* fed on calf treated with *B. bassiana* was slightly higher than on untreated calf (*χ*_1_^2^ = 7.64, *P* = 0.01, Figure 3(a)). However, the magnitude of proportion of fed mosquitoes was >0.93 in both cases (Figure 3(a)).

**(ii) Proportion of Fungus-Infected Mosquitoes**

A freshly fungal-treated calf infected a significantly higher proportion of fed *An. arabiensis* than 3-days posttreated calf (*χ*_2_^2^ = 101.53, *P* < 0.001, Figure 3(b)). Calves infected a proportion of 0.90 of fed *An. arabiensis* immediately after treatment, and ~0.70, 3 d after treatment (Figure 3(b)).

**(iii) Effect of Fungus on Mosquito Survival and Fecundity**

The infection of semifield *An. arabiensis* with *B. bassiana* significantly reduced their subsequent number of eggs and survival (Figures 3(c) and 3(d)). The number of eggs laid by *An. arabiensis* fed on fungal-treated calf was 17 eggs less than on untreated calves (*χ*_1_^2^ = 5.17, *P* = 0.02, Figure 3(c)). Similarly, the postfeeding survival of fed *An. arabiensis* varied significantly between fungus-treated and untreated calves (*χ*_1_^2^ = 19.3, *P* < 0.001, Figure 3(d)). The risk of death (hazard ratio-HR) of fed *An. arabiensis* on fungus-treated calves was almost twice that on untreated calves (HR = 1.63 (1.31–2.03), Figure 3(d)).

### 3.2. Small-Scale Field Experiments

**(i) Proportion of Fed Wild Mosquitoes**

**Figure 4:**
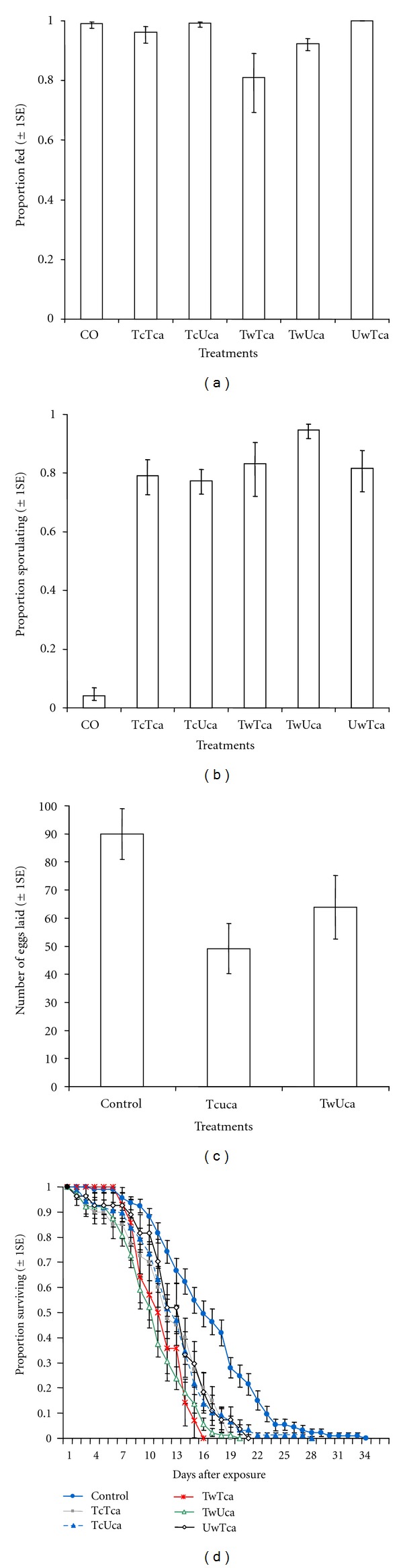
Effects of *M. anisopliae* IP 46 against wild exophilic *An. arabiensis*. (a) Estimated proportion (±  1s.e ) of fed mosquitoes after exposure to fungus-treated and untreated calf inside experimental hut. (b) Estimated proportion (±  1s.e) of infected mosquitoes after exposure to fungus-treated and untreated surfaces. (c) Estimates (±  1s.e) of the mean number of eggs laid by mosquitoes after exposure to fungus-treated and untreated surfaces. (d) Survival of mosquitoes after exposure to fungus-treated and untreated surfaces. The lines represent the survival function as estimated from the fitting Cox proportional hazard model (controlling for random variation between individual calves).

The proportion wild *An. arabiensis* fed on their natural preferred host (calf) ranged from 0.81 to 1 under field conditions. The proportion of fed wild *An. arabiensis* from control was similar to all other treatments (*P* > 0.05, Figure 4(a)). Similarly, within treatments, two-way comparisons revealed that no significant differences in proportion of fed wild *An. arabiensis* between treatments (*P* > 0.05, Figure 4(a)).

**(ii) Proportion of Fungus-Infected Mosquitoes**

The proportion of wild *An. arabiensis* infected with *M. anisopliae* isolate IP 46 varied significantly between delivery methods (*χ*_5_^2^ = 228.05, *P* < 0.001, Figure 4(b)). The multiple comparisons revealed that the proportion of infected mosquitoes observed in control was significantly lower than all treatments: treated (cloth + calf), TCTca (*z* = 7.18, *P* < 0.001, Figure 4(b)), treated cloth + untreated calf, TCUca (*z* = 7.78, *P* < 0.001, Figure 4(b)), treated (wall + calf), TwTca (*z* = 5.73, *P* < 0.001, Figure 4(b)), treated wall + untreated calf, TwUca (*z* = 8.72, *P* < 0.001, Figure 4(b)), and untreated wall + treated calf, UwTca (*z* = 6.68, *P* < 0.001, Figure 4(b)). Within fungal treatments, the proportion of infected mosquitoes was observed to differ significantly between TCUca and TwUca (*z* = 3.25, *P* = 0.01, Figure 4(b)).

**(iii) Effect of Fungus on Mosquito Survival and Fecundity**

The infection of mosquitoes with *M. anisopliae* significantly reduced the number of eggs laid by wild *An. arabiensis* (*χ*_2_^2^ = 13.61, *P* = 0.001, Figure 4(c)). From pairwise multiple comparisons, mosquitoes from the control hut laid significantly more number of eggs than those from treated cottoncloth +  untreated calf, TCUca, (*z* = 4.98, *P* < 0.001, Figure 4(c)) and treated walls + untreated calf, TwUca (*z* = −2.43, *P* = 0.04, Figure 4(c)). However, there was no significant differences between the number of eggs laid by mosquitoes from the hut with TCUca and TwUca (*z* = 1.54, *P* = 0.27, Figure 4(c)).

The infection of *An. arabiensis* with *M. anisopliae* had also significantly affected their longer-term survival (*χ*_5_^2^ = 83, *P* < 0.001, Figure 4(d), Table 1). The risks of death (hazard ratio) of these mosquitoes were significantly lower on untreated surfaces than on fungus-treated surfaces (TCTca: *χ*_1_^2^ = 22.2, *P* < 0.001, TCUca: *χ*_1_^2^ = 29.9, *P* < 0.001, TwTca: *χ*_1_^2^ = 22.0, *P* < 0.001, TwUca: *χ*_1_^2^ = 73, *P* < 0.001, UwTca: *χ*_1_^2^ = 13.6, *P* < 0.001, Figure 4(d), Table 1). However, no significant differences of the hazard ratio of fed *An. arabiensis* were observed across fungal treated surfaces (*P* > 0.05, Figure 4(d)).

## 4. Discussion

This study demonstrates for the first time the potential of applying fungus to cattle and their milieu (mud walls and roofs) for controlling wild population of exophilic *An. arabiensis*. A high proportion of these mosquitoes was strongly attracted and fed on cattle (>0.90) and became infected with fungus treatments. Notably, fungus-treated mud walls infected a higher proportion of mosquitoes (~0.94) than treated calf and cotton-cloth roof or their combinations under field conditions. The fecundity of fungus-infected mosquitoes on calf, mud wall and cotton-cloth roof was, respectively, 17, 40, and 27 eggs less than those on control. Surprisingly, the effects of fungus on survival of *An. arabiensis* were similar between delivery methods and/or their combinations. However, the magnitude of relative risk of death of mosquito on fungus-treated mud walls was 2 times more than that on treated calf and cotton-cloth roof. Therefore, cattle milieu, mostly mud walls of their houses, could be the best field delivery method of fungus against a population of exophilic, zoophilic *An. arabiensis*, suggesting a bio-insecticide zooprophylaxis.

Entomopathogenic fungi, notably *Metarhizium anisopliae* and *Beauveria bassiana,* are promising potential bio-insecticide against malaria vectors [23, 37, 50]. The mud walls treated with *M. anisopliae* IP 46 infected a higher proportion of wild *An. arabiensis* (0.94) than all other treatments or their combinations. This exophilic *An. arabiensis *is generally known to feed on cattle or on humans if available [5, 7]. Therefore, cattle attracted a high proportion (>0.90) of these mosquitoes that became fed and rest on fungus-treated mud walls because the oil formulation of fungal conidia is nonrepellent [25]. Besides, the four sides of the hut provided a bigger surface area of fungus exposure than a calf, and therefore, the likelihood of mosquitoes picking up more conidia on mud walls than on a calf. Furthermore, mud walls provided a natural medium (soil) for better fungal conidia attachment and viability. These results support the previous studies that demonstrate when fungal conidia applied on soil medium (e.g., mud panels [52], clay pots/tiles [19, 36]) the infection rates are higher relative to other substrates. One of these studies demonstrated that a humid and cool environment in a clay pot potentiates high infection rates (>92%) in *An. gambiae* s.s and *An. funestus* in the laboratory and suggested that a human synthetic odour should be incorporated to maximize the number of mosquitoes exposed to fungal conidia [36]. The present study demonstrated that natural preferred hosts (cattle) could attract a high proportion of exophilic *An. arabiensis* that become infected with fungus applied on mud walls of cattle house 

 Unexpectedly, a significantly low proportion (~0.04) of fed wild *An. arabiensis* was infected by fungus treatments. The possible explanation for this observation could be that fed mosquitoes from fungus-treated huts flew into control hut while seeking for the resting places after blood feeding. Alternatively, mosquitoes could have been drifted by wind from fungus-treated huts to control hut.

The fungus is a nonrepellent [25], slow acting bio-insecticide [24], that allows mosquitoes to feed, rest on treated surfaces while developing eggs (e.g., for *Anopheles* mosquitoes take 3-4 days to develop eggs after blood feeding, [46]), and subsequently lay eggs before being killed. This study demonstrated that fungus-infected *An. arabiensis* laid 17–40 eggs less than those mosquitoes in the control group. The possible explanation could be that fungus and mosquitoes compete on the same protein resources and therefore fed mosquitoes that became infected allocated these resources on their survival than egg development. The observations in this study are consistent with those reported elsewhere [19, 31]. Although a laboratory study revealed that no statistical significant differences between the numbers of eggs laid by fungus-infected and noninfected mosquitoes [53], this is largely linked with similar blood meal size between these two groups such that whatever depleted by fungus left these mosquitoes with threshold volume of blood meal required for egg development [54, 55].

Evolutionary forces act upon reproduction and survival of malaria vectors. The opportunity of surviving fungus infection to lay at least few eggs suggests that they are less likely to generate selection pressures for resistance [24]. The daily survival rates of *An. arabiensis* were significantly reduced by fungus-treated surfaces by >2 times than on control in both semifield and field conditions. However, the magnitude of the relative risk of death of mosquitoes on fungus-treated mud wall was consistently 4 times higher than on control. These findings are consistent with previous studies that have shown that fungus-treated surfaces reduce daily survival rates of *An. gambiae s.l* more than untreated surfaces [29, 37]. Similarly, the observations that the efficacy of fungus against mosquitoes was high when applied on mud walls are consistent with those reported under laboratory conditions on mud tiles [19] and mud panels [52]. Most importantly, the present study demonstrated that fungus-treated mud walls killed ~70% of the wild population at day 11 after exposure, suggesting that this option has potential of interrupting transmission of *Plasmodium falciparum* that requires 14 days to develop to transmissible sporozoites within mosquitoes [30].

Fungus kills mosquitoes in 4–10 days depending on exposure dose, viability and virulence of the fungal species/strain, and physiological status of mosquitoes [19, 49, 56]. Therefore, the results reported here have limitations before being compared with other studies or translated into application. First, the present study reported the effect of fungus infection on daily survival rates of blood fed mosquitoes (>95% blood fed mosquitoes) and therefore cannot be directly compared with nonblood fed mosquitoes in other studies. Previous studies have shown that blood resources improve daily survival rates of mosquitoes [57–59], and fungus infection kills nonblood fed mosquitoes much faster than blood fed ones [49]. Second, we briefly measured persistence of fungal conidia on calf under semifield conditions. This study found that ~70% of semifield reared *An. arabiensis* could be infected by a 3-days-post treated-calf. However, detailed experiments on persistence of fungal conidia on cattle and mud walls are now planned to be conducted under field conditions: (1) to test for the effect of sunlight (UV) and rainfall on persistence of fungal conidia in grazing cattle, (2) to test for the effect of wet and dry season or smoke on the persistence of fungal conidia on mud walls. Third, this is the first study to apply fungus on cattle for the control of malaria vectors, and therefore we could have underdose mosquitoes. We sprayed the whole body of a calf with a fungal conidia dose of 1.0 − 2.2 × 10^9^ conidia mL^−1^ which was slightly higher than the dose tested (1 × 10^8^ conidia mL^−1^) on cattle to control ticks [60, 61]. However, ticks are sticking on cattle body for days while feeding until become engorged whereas mosquitoes fly and land on cattle body to feed for few minutes (temporary ectoparasites). This suggests that mosquitoes might have been underdosed. The full-field experiments have been designed to test different doses of fungal conidia applied on either mud walls or calf against wild *An. arabiensis*. Fourth, the use of different fungus species in these experiments: *B bassiana* for semifield experiments and *M. anisopliae* IP 46 for small-scale field experiments. The intention was not to compare their efficacy but was because of the availability of conidia. Although the two fungus species showed similar trend in the semifield and field conditions, now experiments are underway using *M. anisopliae* IP 46 and *B. bassiana* at the same time in the field to confirm if they act on the same direction against malaria vectors in the natural environments.

This study demonstrates for the first time the potential of applying fungus on cattle and their milieu (mud walls and cotton-cloth roof) for the control of exophilic wild *An. arabiensis*. A high proportion of exophilic *An. arabiensis* was attracted to both fungus-treated and untreated cattle where they fed and became infected by different fungus treatments. The fungus-treated mud walls infected a higher proportion of *An. arabiensis* than all other treated surfaces and their combinations. Surprisingly, fecundity and survival of infected mosquitoes were similar between treatments and their combinations, but varied from their controls. Although not significantly but the magnitude of risk of death of these mosquitoes on fungus-treated mud walls was 2 times more than on treated calf and cottoncloth roof. These results suggest that a combination of fungus-treated mud walls and untreated cattle in their milieu could be acceptable, cheap, and easy to apply in rural settings, thus making a perfect bio-insecticide zooprophylaxis that may compliment universal coverage of LLIN.

## Figures and Tables

**Table 1:** Hazard ratio of *An. arabiensis* after exposure on different fungal-treated surfaces and cattle. The numbers in brackets are 95% confidence intervals. The treatments are abbreviated as TCTca for (treated cloth roof + calf), TCUca for treated cloth + untreated calf, TwTca for (treated mud wall + treated calf), TwUca for (treated mud wall + untreated calf), and UwTca for (untreated mud wall + treated calf).

Fungal treatments	Hazard ratio (HR) Relative to control
Treated (cloth + calf)	2.56 (1.73–3.78)
Treated cloth + untreated calf	2.36 (1.73–3.21)
Treated (wall + calf)	4.05 (2.26–7.26)
Treated wall + untreated calf	4.13 (2.99–5.71)
Untreated wall + Treated calf	2.30 (1.48–3.58)
